# Predictive Value of Pelvic Floor Muscle Morphometry Using 3D/4D Ultrasound in Relation to the Success of Pelvic Floor Muscle Training in Women with Stress Urinary Incontinence

**DOI:** 10.3390/ijerph192214757

**Published:** 2022-11-10

**Authors:** Magdalena Hagovska, Ján Svihra, Peter Urdzik

**Affiliations:** 1 Department of Physiatry, Balneology, and Medical Rehabilitation, Faculty of Medicine, Pavol Jozef Safarik University, 040 01 Kosice, Slovakia; 2 Urogynecology and Physiotherapy in Gynecology and Urology, Clinic Centrum s.r.o., 040 01 Kosice, Slovakia; 3 Department of Urology, Jessenius Faculty of Medicine in Martin, Comenius University Bratislava, 814 99 Bratislava, Slovakia; 4 Department of Gynecology and Obstetrics, Faculty of Medicine, Pavol Jozef Safarik University, 040 01 Kosice, Slovakia

**Keywords:** pelvic floor exercise, stress urinary incontinence, prediction, pelvic floor muscle morphometry

## Abstract

The aim of our study was to establish the predictive value of pelvic floor muscle morphometry using 3D/4D ultrasound in relation to the success of pelvic floor muscle training (PFMT) for 12 weeks in women with stress urinary incontinence (SUI). A total of 86 women with SUI from regional gynaecological and urological outpatient clinics were enrolled on this cross-sectional study. SUI symptoms were assessed by the International Consultation on Incontinence Questionnaire (ICIQ-UI SF). Pelvic floor muscle function was evaluated using a perineometer. Pelvic floor muscle morphometry (PFMM) was evaluated by the size of the urogenital hiatus (HA in cm^2^) at rest (R), at contraction (C) and during the Valsalva manoeuvre, i.e., a strong push (V), by 3D/4D USG. The intervention was PFMT for 12 weeks. After PFMT, we noted significant improvement in SUI symptoms, pelvic floor muscle function and morphometry. Moderately significant (0.001) negative correlations were confirmed between the total ICIQ-UI SF score and strength (−0.236 **) and endurance (−0.326 **) of the maximal voluntary contraction (MvC), the number of MvC lasting 3 s (−0.406 **) and 1 s (−0.338 **). Moderately significant (0.001) positive correlations were confirmed between the total ICIQ-UI SF score and R (r = 0.453 **), C (r = 0.533 **) and V (r = 0.442 **). The predictive value of PFMM reached a positive prediction of a decrease with an ICIQ-UI SF score below 8. HA during V was most strongly associated with SUI reduction, with an area under the curve (AUC) of 0.87 (*p* ≤ 0.001), a positive predictive value of 83.3%, a negative predictive value of 75.0%, sensitivity of 78.9% and specificity of 80.0%. The predictive values of pelvic floor muscle morphometry using 3D/4D USG confirmed the success of PFMT in women with SUI.

## 1. Introduction

Stress urinary incontinence (SUI) is defined as a complaint of involuntary leakage of urine during physical activity or sports, sneezing or coughing due to increased intra-abdominal pressure [1]. According to the International Urogynecological Society (IUGA) and the International Society for Continence (ICS), pelvic floor muscle training (PFMT) is the first method of treatment for stress urinary incontinence (SUI) in women [1].

Before the start of PFMT, we need to examine pelvic floor muscle function. It is important to examine the pelvic floor muscle tone and the occurrence of pain. Subsequently, the strength, endurance and relaxation of the pelvic floor muscles, as well as the way of pelvic floor muscle activation and stereotypical breathing should be examined. It is also important to examine the posture and the superficial muscle structures in the area of the pelvis, spine and lower limbs [2,3].

The examination of pelvic floor muscle morphometry using 3D/4D ultrasound (USG) is also very important. It is possible to visually observe the m. puborectalis muscle complex at rest, during contraction, during the Valsalva manoeuvre and while coughing. The 3D USG images are static in three-dimensional space, while 4D USG is 3D USG over time. This examination can quantify the function of the pelvic floor muscles. On 2D USG, we can see the anorectal angle and the levator plate angle, and we can measure the distance between the symphysis and the anorectal angle. However, we cannot visualise the hiatal space [4].

Ultrasound helps to visualise the function of the pelvic floor muscles, and to assess the tonus of pelvic floor (PF) muscles by measuring the size of the hiatal space; it can also be used as biofeedback. The perineometer shows the strength and endurance of the PF muscles in numerical values and can also be used as biofeedback. According to these findings, it is important for patients to undergo targeted PFMT, taking into account the results of this examination.

In patients with SUI, it is most often necessary to train the pelvic floor muscles regarding both strength and endurance. It is important to learn how to properly control the pelvic floor muscles [1,2,3,4].

Pelvic floor muscle morphometry has been used in several studies. Bø et al. [5] examined the correlation between vaginal resting pressure and pelvic floor muscle strength and endurance and hiatal space in pregnant nulliparous women. Albrich et al. [6] investigated the correlation between 2D and 3D USG examination and pelvic floor muscle contractility in women. However, these studies did not establish the predictive value of pelvic floor muscle morphometry using 3D/4D ultrasound in relation to pelvic floor muscle training.

The aim of this study was to establish the predictive value of pelvic floor muscle morphometry using 3D/4D ultrasound in relation to the success of PFMT with stabilisation for 12 weeks in women with SUI.

## 2. Materials and Methods

### 2.1. Study Design

This interventional single-arm study was conducted between May 2020 and January 2022 in the Urogynaecology and Physiotherapy in Gynaecology and Urology clinics, at Clinic Centrum. All the included probands provided signed informed consent. The study was approved by the local ethics committee with the number 3545/2020/ODDZ-06621 on 28 February 2020.

### 2.2. Data Collection

**Figure 1:**
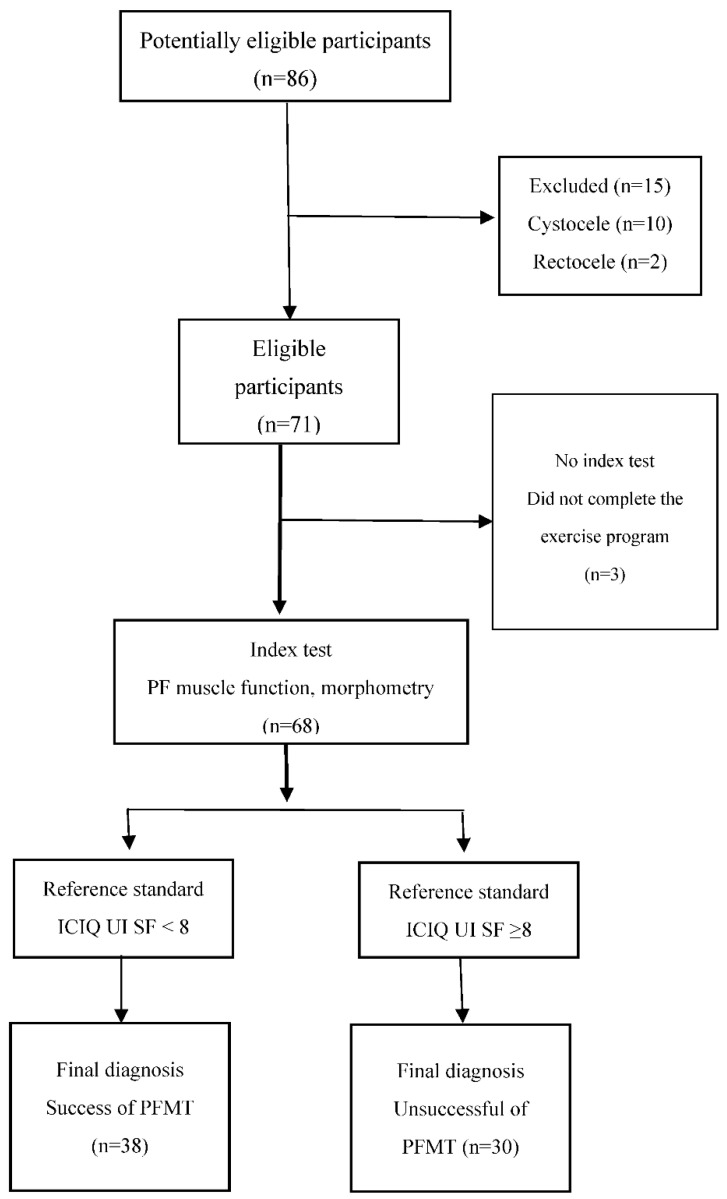
STARD diagram.

Women with SUI from regional gynaecological and urological outpatient clinics were recommended for PFMT by a physiotherapist. In total, 86 women were enrolled in the study and provided signed informed consent. Based on the inclusion and exclusion criteria, 15 women were excluded (ten women had cystocele, two women had rectocele and three women had urethral surgery within three months of the beginning of the study). Three women did not complete the exercise program. The final sample consisted of 68 women (Figure 1).

### 2.3. Sample Size Calculation

We used power analysis to determine the appropriate sample size for a one-sample *t*-test; power 0.80, alpha 0.05 (type I error). The sample size was calculated for a 30% improvement in stress urinary incontinence by the ICIQ-UI SF moderate score (6–12). Based on these assumptions, a minimum sample size of 60 women was estimated. We expected a 30% loss, so we included a total of 86 women.

### 2.4. Inclusion Criteria

The inclusion criteria were as follows: 1. Women willing to provide written informed consent. 2. Women over 18 years of age experiencing uncomplicated SUI. 3. Score on the International Consultation on Urinary Incontinence Questionnaire ≥ 6 points. 4. Symptoms of urinary incontinence for at least 3 consecutive months. 5. Degree of pelvic organ prolapse, stage ≤ 2.

### 2.5. Exclusion Criteria

The exclusion criteria were as follows. Complicated SUI refers to SUI associated with:

1. History of anti-incontinence surgery in the past 12 months.
2. History of pelvic prolapse repair or urethral surgery in the past 12 months.
3. History of pelvic irradiation.
4. Presence of voiding symptoms or neurogenic lower urinary tract (LUT) dysfunction or significant associated overactive bladder (OAB).
5. History of PFMT in the past 12 months.
6. History of interstitial cystitis or bladder-related pain.
7. Chronic severe constipation.
8. Clinically significant renal or hepatic impairment.
9. Clinically significant heart impairment.
10. Pregnant, lactating or actively trying to become pregnant.
11. Presence of urinary tract infection.
12. Use of rehabilitation aids (pessary, urethral plugs, vaginal beads, etc.).
13. Insufficient understanding of pelvic floor exercises and/or omitting exercises.
14. Incomplete questionnaire.
15. Refusal to participate in the study [7].

### 2.6. Procedures

#### Intervention Program—Pelvic Floor Muscle Training with Stabilisation Exercises for 12 Weeks

1. Education of the proband about the anatomy, physiology and function of the pelvic floor muscles, about the correct posture and understanding of the exercise.
2. Training of pelvic floor muscles in various positions, i.e., lying on the back, abdomen and side, and while kneeling, sitting, standing and walking.
3. Training of pelvic floor muscles with static and dynamic stabilisation. Respiratory stereotype correction; activation of m. transversus abdominis, mm. multifidi and other deep muscles of the spine of the upper and lower limbs. Strength and endurance of the pelvic floor muscles during the performance of a bridge by lifting the pelvis while lying on the back with the legs bent; during diagonal stabilisation in the kneeling position with a stretched forearm of the right hand and extending the left leg backwards; during various forms of push-ups, i.e., at the wall, at the table, man and woman push-up; during squats and squatting on one leg and during the execution of a side bridge. Dosage: 12 weeks, on average 3 times a week for 20 min a day, 5 times with training by a physiotherapist, followed by continuation in the home environment.

### 2.7. Primary Measurements

Urine leakage symptoms were assessed using the International Consultation on Incontinence Questionnaire, short form (ICIQ-UI SF), developed by the International Continence Society (ICS). It monitors the frequency and amount of urine leaked in the first two questions. The third question looks at how much urine leakage affects patients’ daily lives. The ICIQ-UI SF score is the sum of the three questions. The severity of UI according to the ICIQ-UI SF is as follows: slight (1–5), moderate (6–12), severe (13–18) and very severe (19–21). Cronbach’s alpha of the ICIQ-UI SF is 0.95 [8,9].

The cut-off point was determined according to the median of the final ICIQ-UI SF score. A value of the ICIQ-UI SF score below the cut-off point indicated the success of PFMT treatment and a reduction in SUI symptoms. The score of the ICIQ-UI was the reference standard of analysis.

The Overactive Bladder Questionnaire—symptom score (OAB-q SS) focuses on the symptoms of an overactive bladder in the last 4 weeks. It contains six questions on symptoms, giving the symptom score (0—without symptoms; 100—most symptoms). Cronbach’s alpha is 0.90 [10,11]. The symptom score OAB-q SS was considered insignificant if it reached a value of less than 20.

### 2.8. Secondary Measurements

#### 2.8.1. Examination of Pelvic Floor Muscle Function

A perineometer (Peritron, Ontario, L4 V, Canada) was used to quantify maximal voluntary contraction (MvC) in units of water column height in cmH_2_O, the endurance of MvC in seconds, repeated MvC (3 s) and fast MvC (1 s), and relaxation of the pelvic floor muscles. The pressure probe was 11 cm in length (25–90 mm) and covered with a latex condom to which a lubricant was applied. The examination was performed with an empty bladder in a lithotomic position (i.e., lying on the back). The probe was inserted vaginally without pain or discomfort. The phase of the menstrual cycle was recorded. Up to 10 cmH_2_O indicates weak contraction, 10–30 cmH_2_O indicates moderately strong contraction and 40–60 cmH_2_O indicates strong contraction [12].

#### 2.8.2. Examination of Pelvic Floor Muscle Morphometry Using 3D/4D USG

The examination was performed with a Voluson-i BT 11 Console, VCI volume contrast imaging software (GE Healthcare Austria GmbH & Co. OG, Zipf, Austria) and a probe RAB4-8-RS 3D/4D 4-8 MHz. The examination was performed with an empty bladder in a lithotomic position. The probe was placed longitudinally on the perineum. A 3D/4D image was taken at rest, during MvC and during the Valsalva manoeuvre in cm^2^. The anterior–posterior hiatal dimension and later lateral hiatal dimension were measured in cm. This method was tested for reliability, which was very good [13,14,15]. Examinations were performed by a trained urogynaecologist and physiotherapist.

### 2.9. Statistical Analysis

Descriptive and inferential statistics were used for data analysis. Data are presented as mean values and standard deviation (SD). The Wilcoxon paired non-parametric test was used. The level of statistical significance was set at *p* < 0.05. The degree of the relationship between the variables, e.g., the correlation, was evaluated by the Pearson correlation coefficient. We used the area under the curve (AUC) calculation according to the receiver operating characteristics (ROC). AUC was used to predict SUI to pelvic floor muscle function and morphometry parameters. In the analyses, we used post-treatment measurements. We performed the calculation of predictive values according to the Standards for Reporting Diagnostic Accuracy (STARD) [16]. Index tests were PF muscle function and morphometry. Reference standards were SUI symptoms assessed by ICIQ-UI SF. The cut-off of SUI symptoms was ICIQ-UI SF < 8; mean values of PF muscle function and morphometry MvC in cmH_2_O and endurance of MvC in seconds, as well as mean values of HA at rest, contraction and Valsalva manoeuvre in cm^2^ after 12 weeks of PFMT are reported (Figure 1). The calculations were performed in IBM SPSS Statistics for Macintosh, Version 28.0. Armonk, NY, USA: IBM Corp.

## 3. Results

The sample consisted of 68 women with SUI with a mean age of 40.4 years; the mean duration of SUI was 23.5 months. The mean number of childbirths was 1.7. The mean child weight was 3620.6 g. The mean number of incontinence episodes per week was 8.7. The mean number of pads used per day was 1.2. The mean ICIQ-UI SF score was 9.8, which indicates a moderate level of SUI symptoms. The OAB-q SS score was 7.9, which indicates non-significant OAB symptoms. The cut-off point was determined according to the median of the final ICIQ-UI SF score, where a value lower than 8 indicates the success of PFMT treatment with a reduction in SUI symptoms. The score of the International Consultation on Urinary Incontinence Questionnaire was the reference standard of analysis. Muscle tone was normal. Pelvic floor muscle prolapse was not present. According to the OAB-q SS questionnaire, we excluded women with symptoms of urgency urinary incontinence (Table 1).

We compared the baseline and final median of the ICIQ-UI SF score, the number of pads/day, pelvic floor muscle function and morphometry. After treatment, we noted significant improvements in SUI symptoms, with a decrease in the ICIQ score and a decrease in the number of pads/day; 15 (22.1%) women had no leakage, 6 (8.8%) women had slight SUI, 40 (58.8%) women had moderate SUI and 7 (10.3%) women had severe SUI.

We found significant differences in the improvement of pelvic floor muscle function in MvC, endurance of MvC, repeated MvC and fast MvC. We also found significant differences in the hiatus reduction in cm^2^ during rest, contraction and the Valsalva manoeuvre.

Before treatment, no significant correlations were found between ICIQ-UI SF and pelvic floor muscle function. Only two weak significant correlations were confirmed between ICIQ-UI SF and urogenital hiatal area at rest (P; r = 0.248 *), as well as contraction (C; r = 0.283 *).

After treatment, moderately significant (0.001) negative correlations were confirmed between the reduction in the total ICIQ-UI SF score and the increase in the strength (−0.236 **) and endurance (−0.326 **) of the MvC, as well as the number of MvC lasting 3 s (−0.406 **) and 1 s (−0.338 **) by the perineometer. This means that improved parameters of pelvic floor muscle function were associated with a reduction in symptoms of urine leakage (Table 2).

Moderately significant (0.001) positive correlations were confirmed between the reduction in total ICIQ-UI SF score and pelvic floor muscle morphometry: a reduction in the size of the urogenital hiatal area at rest (P; r = 0.453 **), contraction (C; r = 0.533 **) and the Valsalva manoeuvre (V; r = 0.442 **) by 3D/4D ultrasound. This means that, with a smaller hiatal area at rest, during contraction and the Valsalva manoeuvre, the symptoms of urine leakage were reduced (Table 3).

**Figure 2:**
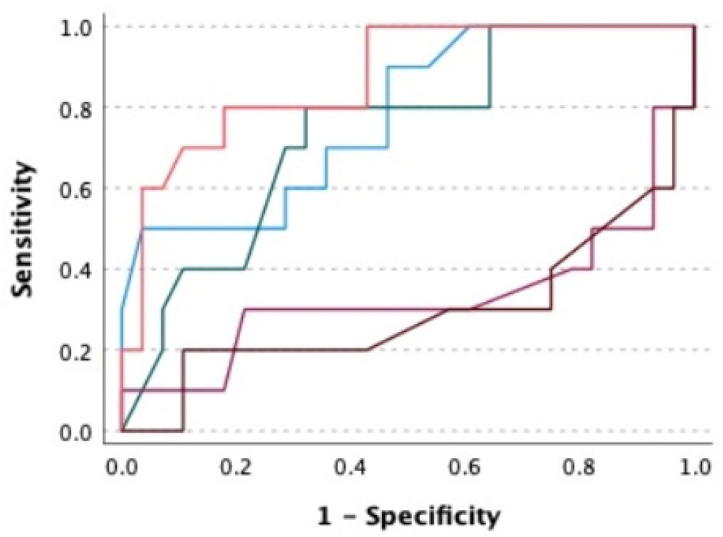
Area under the curve prediction of SUI according to pelvic floor muscle function and morphometry. Legend: HA—urogenital hiatal area in cm^2^ according to 3D/4D ultrasound. Maximal voluntary contraction (MvC) and Endurance of MvC in seconds. Blue line: HA during contraction (cm^2^). Green line: HA at rest (cm^2^). Pink line: HA during the Valsalva manoeuvre (cm^2^). Purple line: MvC (cmH_2_O). Brown line: endurance of MvC (seconds).

The ROC expresses the prediction of SUI development according to individual parameters of pelvic floor muscle function and morphometry. HA during the Valsalva manoeuvre was most strongly associated with a reduction in SUI, according to the ROC analysis, with an AUC of 0.87. The HA at rest and during contraction values were smaller but strongly associated with a reduction in SUI, according to the ROC analysis, with an AUC of 0.74–0.78. Pelvic floor muscle morphometry positively predicted a reduction in the ICIQ-UI SF score below 8. MvC and its duration did not indicate a positive prediction of a decrease in ICIQ-UI SF to a score below 8 (Table 4, Figure 2).

The predictive values of pelvic floor muscle morphometry using 3D/4D USG confirmed the success of PFMT in women with SUI (Table 5).

## 4. Discussion

The aim of this study was to establish the predictive value of pelvic floor muscle morphometry using 3D/4D ultrasound in relation to the success of PFMT with stabilisation for 12 weeks in women with stress urinary incontinence.

After treatment, we noted significant improvements in SUI symptoms, indicated by a lower ICIQ score and decrease in the number of pads/day; 15 (22.1%) women had no leakage, 6 (8.8%) women had slight SUI, 40 (58.8%) women had moderate SUI and 7 (10.3%) women had severe SUI. Despite significant improvement in SUI symptoms, not all women achieved an adequate clinical reduction of SUI symptoms. We believe that if the intensity of the exercise could be increased from 3 times a week for 20 min a day, e.g., to 6 times a week, we would notice a more significant reduction in SUI symptoms. We noted significant improvements in pelvic floor muscle function with stronger maximal voluntary contraction and longer duration. Improvements were also seen in pelvic floor muscle morphometry, interpreted as a reduction in hiatal area in cm^2^ during rest, contraction and the Valsalva manoeuvre.

Correlations between SUI symptoms and pelvic floor muscle function and morphometry after PFMT were confirmed. A stronger MvC and longer duration were associated with a greater reduction in the symptoms of urine leakage. A smaller hiatal area at rest, during the contraction and the Valsalva manoeuvre, was also associated with a greater reduction in the symptoms of urine leakage.

The size of the hiatal area during the Valsalva manoeuvre was most strongly associated with a reduction in the symptoms of SUI, according to the ROC analysis. The hiatal area at rest and during contraction was less but strongly associated with a reduction of symptoms of SUI, according to the ROC analysis. Pelvic floor muscle morphometry positively predicted a decrease in the ICIQ-UI SF score below 8. According to the reported findings, pelvic floor muscle morphometry with 3D/4D USG should be an important part of the diagnosis and treatment of women with SUI. Each diagnostic method, as well as USG, requires years of experience and adequate training, which the therapists in our study had.

The average age of women in our population was around 40 years. It would be appropriate to carry out similar measurements in older women as well as in women with hypotonicity of the pelvic floor muscles.

There have been several studies that evaluated pelvic floor muscle morphometry in relation to physiotherapy. Bø et al. [5] examined the correlation between vaginal resting pressure, pelvic floor muscle strength, endurance and levator hiatus area in 300 pregnant nulliparas. Strong pelvic floor muscle contraction correlated slightly with a smaller levator hiatus area. Increased resting tone correlated with decreased levator hiatus area. However, this study was not interventional and pelvic floor muscle morphometry was not evaluated after the intervention. In our study, the hiatal area during the Valsalva manoeuvre was most strongly associated with a reduction in SUI, according to the ROC analysis after 12 weeks of PFMT.

Albrich et al. [6] assessed the correlation between 2D/3D ultrasound examination and pelvic floor muscle contractility in 114 women. Here, positive correlations were found between bladder neck elongation and a reduction in the symphysis–levator distance, and a reduction in the levator hiatus area and palpable vaginal examination of muscle strength. In our study, correlations between SUI symptoms and pelvic floor muscle function and morphometry after PFMT were confirmed. Better parameters of pelvic floor muscle function were associated with fewer symptoms of urine leakage.

Cacciari et al. [17] evaluated pelvic floor muscle morphometry and function immediately after 12 weeks of intensive pelvic floor muscle training and after a 12-month follow-up in 362 elderly women with urinary incontinence. Immediately after 12 weeks, significant improvements in pelvic floor muscle morphometry were observed when coughing and during contraction. These improvements persisted after a year of exercise. While coughing, the pelvic floor structures were firmer, there were fewer caudal movements of the puborectal sling and less enlargement of the hiatus during the cough. The PF muscles were stronger, in better coordination, and had better endurance. In our study, after 12 weeks of PFMT, we similarly observed less enlargement of the hiatus at rest, during contraction and during the Valsalva manoeuvre.

### Strengths and Limitations

The strengths of the study are the objective measurement of the pelvic floor muscle function using a perineometer, the utilisation of pelvic floor muscle morphometry by 3D/4D ultrasound and the use of standardised measuring tools, including ICIQ-UI SF. The limitations are that this was not a multicentre study.

Another limitation is that the average age of women in our population was around 40. Therefore, the results in older age groups could be different.

A longer follow-up of the treatment effect, for example after 1 year, would be appropriate in order to confirm the long-term effect of exercise.

## 5. Conclusions

The reduction in SUI symptoms correlates with improvements in pelvic floor muscle function assessed by a perineometer and with pelvic floor muscle morphometry by 3D/4D ultrasound. The predictive value of pelvic floor muscle morphometry using 3D/4D USG confirmed the success of PFMT in women with SUI and positively predicted a decrease in the ICIQ-UI SF score.

PFMT intensity had a partial clinical impact on SUI reduction. Therefore the intensity of PFMT should be increased.

## Figures and Tables

**Table 1:** Demographic characteristics (normal distribution).

n = 68	Mean ± SD/%
Age	40.4 ± 9.1
Education (secondary, university)	49.3%/50.7%
Menstruation (regular/irregular)	83.1%/16.9%
Body mass index (kg/m^2^)	24.1 ± 3.8
Duration of SUI (months)	23.5 ±22.8
Number of childbirth	1.7 ± 0.7
Type of childbirth (no/vaginal/section)	5.6%/83.1%/11.3%
Child weight (g)	3620.6 ± 550.3
Incontinence episode frequency (IEF)/week	8.7± 6.1
Number of pads/day	1.2 ± 1.3
ICIQ-UI SF	9.8 ± 3.1
OABq SS	7.9 ± 8.2

Legend: The International Consultation on Incontinence Questionnaire, short form (ICIQ-UI SF), The Overactive Bladder Questionnaire—symptom score (OAB-q SS).

**Table 2:** Baseline and final percentiles of SUI symptoms, PF muscle function and morphometry and statistical differences before and after treatment (non-parametric distribution).

		Percentiles Baseline	Percentiles Final	
	n = 68	25–50 (Median)–75	25–50 (Median)–75	*p*
**1**	MvC in cmH_2_O (P)	9.00–15.00–22.00	11.10–20.00–35.00	0.001
**2**	Endurance of MvC in seconds (P)	2.00–4.00–6.00	4.00–5.45–10.00	0.001
**3**	Number of MvC 3 s repetitions (P)	1.00–2.00–3.00	3.00–5.00–10.00	0.001
**4**	Number of MvC 1 s repetitions (P)	2.00–4.00–5.00	4.25–10.00–10.00	0.001
**5**	HA—in rest	14.80–16.20–20.30	13.37–15.90–19.55	0.001
**6**	HA—during contraction	12.70–15.50–17.40	11.00–13.80–16.00	0.001
**7**	HA—during Valsalva manoeuvre	15.40–20.20–23.00	14.12–18.00–22.75	0.001
**8**	ICIQ-UI SF	7.00–9.00–12.00	3.25–7.00–9.00	0.001
**9**	Number of pads/day	0–1.00–2.00	0–0–1.00	0.001

Legend: The International Consultation on Incontinence Questionnaire, short form (ICIQ-UI SF), pelvic floor muscle morphometry (PFMM) was evaluated by the size of the urogenital hiatus (HA) in cm^2^ by 3D/4D USG. P—perineometer, maximal voluntary contraction (MvC), the number of MvC lasting 3 s and 1 s. *p*-values—Wilcoxon test.

**Table 3:** The correlation between the ICIQ-UI SF score and pelvic floor muscle function and morphometry before and after treatment.

	Correlations, n = 68	r	*p*	r	*p*
		Baseline		Final	
**1**	ICIQ-UI SF/MvC in cmH_2_O (P)	−0.018	0.08	−0.236	0.001
**2**	ICIQ-UI SF/Endurance of MvC in seconds (P)	−0.154	0.20	−0.326	0.001
**3**	ICIQ-UI SF/Number of MvC 3 s repetitions (P)	0.021	0.862	−0.406	0.001
**4**	ICIQ-UI SF/Number of MvC 1 s repetitions (P)	0.086	0.474	−0.338	0.001
**5**	ICIQ-UI SF/HA—in rest	0.248	0.03	0.453	0.001
**6**	ICIQ-UI SF/HA—during contraction	0.283	0.01	0.533	0.001
**7**	ICIQ-UI SF/HA—during Valsalva manoeuvre	0.121	0.31	0.442	0.001

Legend: r—Pearson correlation coefficient, (P)—according to the perineometer, HA—urogenital hiatal area in cm^2^ according to 3D/4D ultrasound. The International Consultation on Incontinence Questionnaire, short form (ICIQ-UI SF), maximal voluntary contraction (MvC), the number of MvC lasting 3 s and 1 s, *p*-values—Pearson test.

**Table 4:** Area under the curve prediction of SUI according to pelvic floor muscle function and morphometry.

Test Result Variable(s)	Area	*p*	95% Confidence Interval Lower–Upper
HA—in rest in cm^2^	0.74	0.006	0.57–0.91
HA—during contraction in cm^2^	0.78	0.001	0.61–0.94
HA—during Valsalva manoeuvre in cm^2^	0.87	0.001	0.75–0.99
MvC in cmH_2_O (P)	0.44	0.162	0.33–0.56
Endurance of MvC in seconds	0.63	0.064	0.07–0.51

Legend: r—Pearson correlation coefficient, (P)—according to the perineometer, HA—urogenital hiatal area in cm^2^ according to 3D/4D ultrasound, maximal voluntary contraction (MvC).

**Table 5:** Sensitivity, specificity and predictive values of pelvic floor muscle function and morphometry.

n = 68	Sensitivity (%)	95% CI	Specificity (%)	95% CI	Positive Predictive Value (%)	95% CI	Negative Predictive Value (%)	95% CI
HA—in rest	78.95	62.68–90.45	60.00	40.60–77.34	71.43	61.02–79.97	69.23	53.23–81.64
HA—during contraction	73.68	56.90–86.60	70.00	50.60–85.27	75.68	63.56–84.73	67.74	54.01–78.97
HA—during Valsalva manoeuvre	78.95	62.68–90.45	80.00	61.43–92.29	83.33	52.08–73.00	75.00	61.24–85.07
MvC in cmH_2_O	52.63	35.82–69.02	55.00	31.53–76.94	68.97	55.67–79.73	37.93	26.67–50.67
Endurance of MvC in seconds	63.16	45.99–78.19	76.67	57.72–90.07	77.42	63.17–87.27	62.16	50.89–72.26

Legend: r—Pearson correlation coefficient, (P)—according to the perineometer, HA—urogenital hiatal area in cm^2^ according to 3D/4D ultrasound, maximal voluntary contraction (MvC).

## Data Availability

The data presented in this study are available upon request from the corresponding author.
